# Erythema associated with pain and warmth on face and ears: a variant of erythermalgia or red ear syndrome?

**DOI:** 10.1186/1129-2377-15-18

**Published:** 2014-03-26

**Authors:** Ming-Chun Chen, Qing-Fang Xu, Di-Qing Luo, Xiang Li, Ding-Yang He

**Affiliations:** 1 Department of Dermatology, Sun Yat-sen Memorial Hospital, Sun Yat-sen University, 107 Yanjiang Rd. W., Guangzhou 510120, China; 2 Department of Dermatology, The Third Affiliated Hospital, Sun Yat-sen University, 600 Tianhe Rd, Guangzhou 510630, China; 3 Department of Dermatology, The Eastern Hospital of The First Affiliated Hospital, Sun Yat-sen University, 183 Huangpu Rd. E, Guangzhou 510700, China; 4 Department of Stomatology, The Eastern Hospital of The First Affiliated Hospital, Sun Yat-sen University, 183 Huangpu Rd. E, Guangzhou 510700, China

**Keywords:** Erythermalgia, Erythromelalgia, Ear, Face, Pain, Treatment, Variant

## Abstract

Erythermalgia is a rare cutaneous disorder characterized by attacking of erythema, pain and increased temperature, which primarily involves the extremities and may infrequently extend to the neck, face, ears and even the scrotum. We reported an 18-year-old woman who presented with 3 years history of sole involvement of attacking erythema, pain and warmth over her face and ears without any other associations. The frequency and severity of the flares progressed gradually during the course. Cutaneous examination revealed erythema, increased temperature and tenderness on the face and ears during the flare. The symptoms could be relieved rapidly by cooling. Dermatoscope showed that vessels inside the erythema were more dilated during the episode than after application of ice. The lesion is considered a rare variant of erythermalgia with sole involvement of face and ears. The symptoms had mild response to oral antihistamines, topical steroids and tacrolimus, but had excellent response to the combinative therapy of aspirin and paroxetins.

## Background

A few kinds of cutaneous diseases present as attacking facial erythema associated with pain and increased temperature, which mainly include erythermalgia (EM) and red ear syndrome (RES).

EM, first described in 1878 by Mitchell and also termed as “erythromelalgia”, is a rare cutaneous disorder characterized by intermittent redness, increased temperature and pain. Its symptoms may be triggered by warmth or moderate exercise, and can be prevented or relieved by cooling [1-6]. It primarily affects the extremities, particularly the hands and the feet, and may infrequently extend to other parts of the body including the neck, face, ears, nose and scrotum [1,2,7]. In rare instances, localized entity on a thigh [3]; sole involvement on vulva [4], on cheeks [5] and on ears [6] have also been described in the literature. EM includes primary and secondary forms. It is considered that the mutations in SCN9A, encoding the sodium channel protein Na(v)1.7 subunit, are responsible for the primary type [8-10]; while neuropathological and microvascular functional changes may be for the secondary one [8].

RES, first described by Lance in 1994, is characterized by the clinical features mimicking EM. It always occurs in one or less frequently both ears with female predilection and with mean age of 40.2 years, sometimes it affects the adjacent areas including the occipital, forehead or even the jaw [11,12]. Its attacks may be spontaneous, or triggered by touch, exertion, heat or cold stimuli, neck movements, stress, cleaning the ear, etc [11,12]. RES also includes 2 subtypes: the primary and secondary. The primary is an idiopathic form which commonly occurs in young people and associates with migraine, while the secondary form more frequently afflicts the adults associated with cervical disorders and temporo-mandibular joint dysfunction [13].

In the present paper, we describe a woman with sole involvement of attacking erythema associated with pain and warmth on the ears and face, whom we consider is a variant of EM.

## Case presentation

An 18-year-old woman was referred with persistent bilateral erythema, warmth and burning pain over her face and ears. She started the symptoms spontaneously 3 years ago, which attacked several times a month and lasted from 2 to more than 10 hours, even days occasionally. She noticed that the episodes always started on the cheeks and then radiated rapidly over the adjacent areas including ears and forehead without any other associations including scales, sweating and headache. The most painful areas were the cheeks. The symptoms disappeared completely without any other facial lesions between episodes. She was diagnosed as seborrheic dermatitis and was treated with oral antihistamines, topical steroids and tacrolimus, and cool facial masque. The therapeutics seemed to be moderately beneficial, whereas cooling the lesion during the attack usually led to rapid pain relief. During the course, she found that the frequency and severity of the flares progressed gradually. She also noticed that warmth and movement could trigger the attacks or worsen the symptoms while cleaning her teeth, eating, drinking, brushing her hair, touching or rubbing the ears or neck movements had little influence on the attacks; and she preferred to stay in cold environment during the flares since the onset. For the past weeks, she was having constant attacking with nocturnal progress when lying down, which seriously disturbed her quality of life including sleep. The symptoms had poor response to the previously mentioned therapeutics except the cooling face masque. Because of intolerable pain, she had to spray cold water or use cold masque on the lesion all day long. The lesions had never involved the extremities. She had no prior history of migraine, episodic headaches or neck injury. Her family history was also unremarkable.

**Figure 1:**
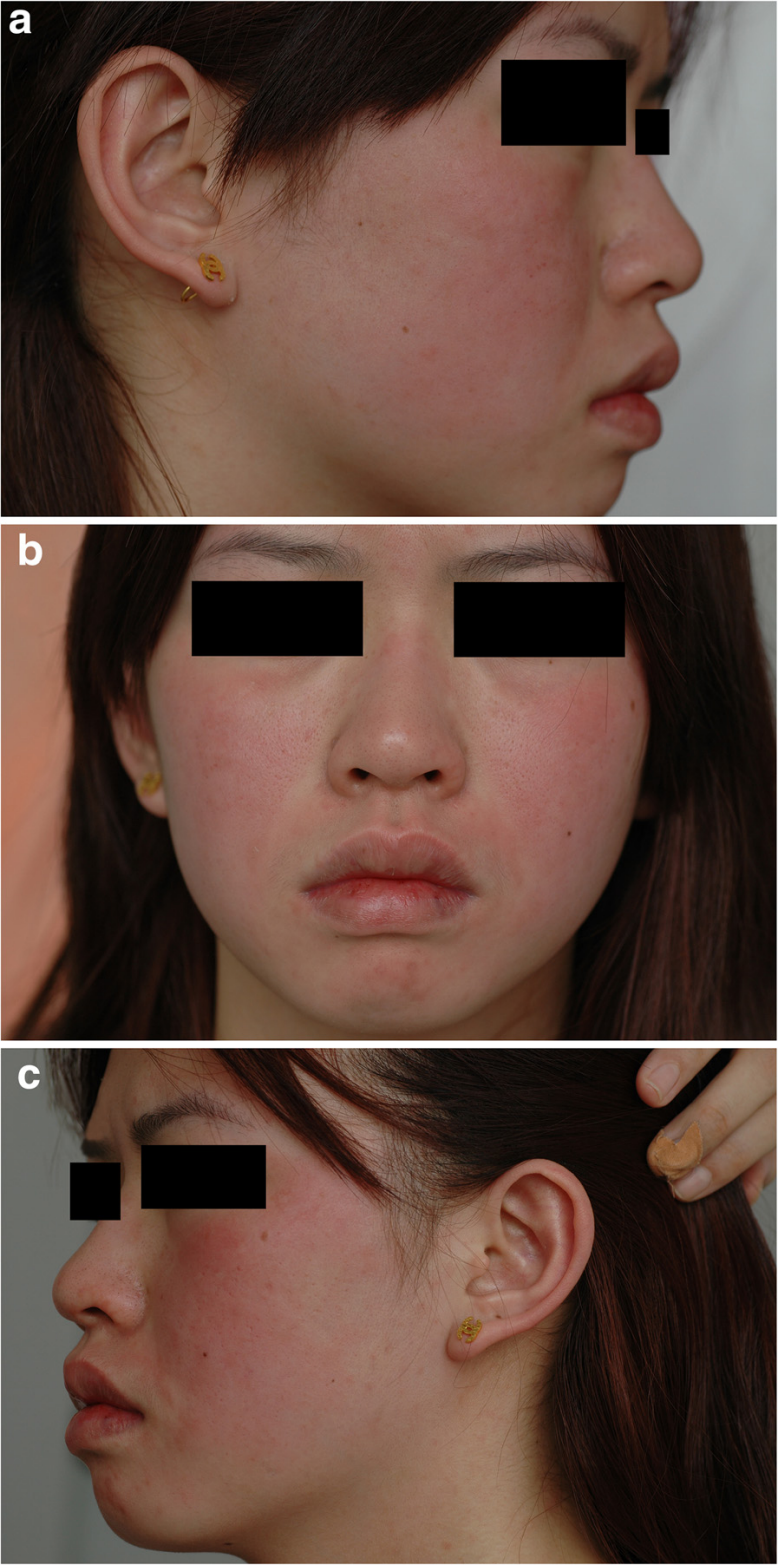
a,b,c Evident erythema on the face and ears during attacking.

On physical examination, she was healthily appearing. During the flare, both ears including the entire helix and antihelix, the forehead and the face were evidently red and tender associated with warmth (Figure 1a-c). The most painful areas were the cheeks. The temperature on the cheek, forehead and axilla was 36.8 degrees centigrade, 36.7 degrees centigrade and 36.8 degrees centigrade, respectively during the attack. She had no swollen plaque over the involved areas and had no lesions on other parts including feet, hands, neck. Dermatoscope showed evidently dilated vessels inside the erythema during the episodes, which decreased markedly after application of ice over the erythema for about 5 minutes. The lesional redness and pain could be alleviated markedly and rapidly by topical application of ice, but resumed rapidly after the removal of ice. Her heart rate and blood pressure were normal during the attacking. Laboratory test for complete blood cell count, chemistry profiles, liver function tests, auto-immune antibodies, antistreptolysin O serology, HIV antibody and TPPA were either within normal limits or negative. The patient refused to take biopsy.

The lesions improved markedly after having been treated with oral gabapentin and indomethacin, and topical lidocaine compounds and cold masque for 3 days; but recurrence occurred 2 weeks after the quit of the treatment. She was then treated with aspirin 0.1, twice a day; paroxetine 10 mg daily, which resulted in complete disappearance of the symptoms after having been treated for 2 weeks. Without any treatment, the patient had been lesion free for 6 months and was still under follow-up.

## Discussion

The present patient had flaring erythema, pain and warmth on her ears and face, and the symptoms lasted for hours even days occasionally and could be aggravated by warmth and relieved by cooling. Although the patient was absent for lesional involvement on extremities, based on the diagnostic criteria [5,14], we considered that the condition was a variant of EM which presented on an atypical location. Considering some EM cases lack limb involvement [4-6] as the present patient occurred, and the origins of the words, we preferred ‘erythermalgia’ rather than ‘erythromelalgia’ to describe such symptoms [5].

EM commonly occurs in the fifth and sixth decades of life with female predilection [1,5]. Interestingly, the case with sole vulva involvement occurred in childhood [4], the patient with sole cheeks affection was a female starting her symptoms at the age of 14 years [5], and the present patient was also a female with an onset at 15 years old, only the case described by Ramirez and Kirsner [6] was a male with an onset of 50 years age. The results revealed that the cases of EM with sole extra-extremity lesions seemed having also female predilection, but with younger age than the typical cases. The reasons for younger age remain unknown, but we cann’t exclude it is just a fortuitous instance as the case number is small. As the dermatoscope of the present case showed more dilated vessels during attacking mimicking the previous one [5], we considered that vascular dilation, at least partially, is responsible for the clinical presentations, although the exact mechanisms are not fully understood.

The differential diagnoses mainly include RES. Although RES and EM share the similar clinical features [5], RES always involves one or less frequently both ears, and rarely the extra-auricular regions. It may also be accompanied by upper cervical disorders, atypical trigeminal and glossopharyngeal neuralgias, temporomandibular joint dysfunction and a thalamic syndrome [11,12], and may be triggered by cleaning teeth, eating, drinking, brushing hair, touching or rubbing the ears or neck movements except warmth [13,15]. Considering the attacking process and triggering factors, we preferred the present case a variant of EM rather than RES. As both EM and RES are similar clinical conditions and share the resemblant diagnostic criteria, for example, the case of auricular EM described by Ramirez and Kirsner [6] strongly resembles RES cases reported by others, and the symptoms of RES reported by Lance mimic that of EM [16], it is indeed hard to distinguish RES from auricular variant of EM sometimes, and some authors even consider that RES may be a variant of EM and both terms describe the similar condition of the ears [17].

Other differential diagnostic considerations for present case are seborrheic dermatitis, relapsing polychondritis, chondrodermatitis nodularis chronica helicis, and especially the facial flushing. As the present patient had attacking erythema associated with increased temperature and pain, and was absent for scales and itching, seborrheic dermatitis can be eliminated. Relapsing polychondritis, presenting with red and swollen ears mimicking our patient, is characterized by intermittent attacks of inflammatory cartilage of the ears and nose. Chondrodermatitis nodularis chronica helicis is characterized by a tender, chronic inflammatory lesion involving the outer helix of the ear, but it also presents with nodules. Both diseases can also be excluded based on the clinical presentations. Based on the spontaneous regression of the lesions and clinical features, the diagnosis of contact dermatitis and photosensitivity can also be ruled out. Facial flushing is usually a symptom of an underlying medical condition or reaction to certain substance, which include alcohol, drugs, allergy, emotions, exercise, food additives, skin disorders, etc. Patients with facial flushing experience a suddenly facial reddening, feel hot face, and always associate with other symptoms relating with the nosogenesis. The reddening may extend to the neck and upper chest, even the whole body, but the patients lack cutaneous pain. Harlequin syndrome is a benign condition showing a sudden onset of unilateral facial flushing and sweating [18], the present case lacked sweating and showed symmetrically facial redness associated with pain and warmth, Harlequin syndrome can also be excluded.

Many therapeutics for EM have been introduced with significant variations in response, no treatment is consistently effective, and to predict the efficacy for a specific treatment is also impossible at present [1-7,14]. Because of its frustrating treatment and management, combinative approaches may be necessary and optimal for EM [1-7,14]. Lumbar sympathetic block was reported in a case showing successful response in a refractory EM recently [19]. Interestingly, the present patient showed excellent response to combinative treatment of aspirin and paroxetine which was rarely reported before.

## Conclusions

EM primarily involves the limbs, and may infrequently extend to other parts of the body including head, neck, and scrotum. But it may solely afflict the cheeks, vulva, ears, or even both face and ears. EM and RES share the similar clinical conditions and diagnostic criteria, it is hard to distinguish RES from auricular variant of EM sometimes. It is possible that both RES and auricular EM may describe the similar condition. Combinative approaches may be optimal for EM. The combination of aspirin and paroxetine is optional therapeutic for such a condition.

## Consent

Written informed consent was obtained from the patient for publication of this Case report and any accompanying images. A copy of the written consent is available for review by the Editor-in-Chief of this journal.

## Abbreviations

EM: Erythermalgia; RES: Red ear syndrom.

## Competing interests

The authors declare that they have no competing interests.

## Authors’ contributions

CMC and XQF conceived of the study, participated in clinical management of the patient, reviewed the literature on the item and drafted the manuscript. LDQ made the correct diagnosis, participated in clinical management of the patient, conceived of the study, reviewed the literature on the item and drafted the manuscript. LX participated the clinical diagnosis, reviewed the literature, drafted and corrected the manuscript. HDY participated in the design of the paper, and drafted the manuscript. All authors read and approved the final manuscript.
